# Means substitutability in personal significance restoration

**DOI:** 10.3389/fpsyg.2023.1193336

**Published:** 2023-07-31

**Authors:** Federico Contu, Molly Ellenberg, Arie W. Kruglanski, Antonio Pierro

**Affiliations:** ^1^“La Sapienza” University of Rome, Rome, Italy; ^2^UniSR-Social.Lab, Vita-Salute San Raffaele University, Milan, Italy; ^3^University of Maryland, College Park, MD, United States

**Keywords:** romantic relationships, work, significance loss, extreme behaviors, motivational imbalance

## Abstract

Drawing on Significance Quest Theory, we hypothesized that when people experience a loss of significance related to a specific life domain, they will aim to restore their significance by acting in an extreme manner in a different life domain. To test this hypothesis, we ran two cross-sectional studies using samples of employed people in romantic relationships. Study 1 tested if people experiencing a loss of significance in the romantic relationship domain were more prone to extremism at work. Study 2 tested whether people experiencing work-related significance loss were more prone to engage in obsessive relational intrusion (ORI) toward their romantic partner. Results from both studies confirmed our hypothesis, suggesting that both amorous relationships and careers are perceived as fruitful in maintaining or restoring ones’ sense of personal significance, even if the original loss of significance is derived from an unrelated domain. Notably, this research represents one of the first tests of the key assumption of Significance Quest Theory entailing the substitutability of means through which one can attain or renew their sense of significance.

## Introduction

In recent years, extreme behaviors between romantic partners have become of central interest within international academic research (e.g., Bélanger et al., 2021). Unfortunately, extremism in romantic relationships seems to be more widespread than one might expect (Cupach and Spitzberg, 2000; Sheridan et al., 2016). As an astonishing example, a recent study found that more than 50% of young adult undergraduate research participants reported having been victims of stalking and intrusive behaviors (Fais et al., 2020). Extremism in other realms is also a concern, including in professional settings. Indeed, more than 10% of the U.S. general population exhibits “workaholism” (Sussman et al., 2011; Andreassen et al., 2012). Such extreme workplace behavior threatens peoples’ personal relationships, mood, and physical health (Griffiths, 2011; Andreassen et al., 2012; Sussman, 2012). It is therefore necessary to understand the psychological processes underlying romantic and professional extremism, and to explore possible relationship between the two.

Extremism of all types is clearly explained by Kruglanski et al. (2021), who define extremism as a situation wherein one psychological need dominates all others, resulting in a motivational imbalance. In a such situation, individuals act in extreme ways, forgoing all other needs, in order to satisfy the dominant need. In the present paper, we identify the quest for significance (Kruglanski et al., 2022) as the universal human need which, when dominant, motivates individuals to enact a slew of extreme behaviors as prescribed by the relevant cultural narrative and social network. Moreover, we explore the possibility that when an individual’s significance need becomes dominant in relation to a particular life domain, they can satisfy that need by acting extremely in another valued life domain. More specifically, we investigate whether when one experiences a loss of significance with respect to a specific life domain (e.g., romantic relationships), they can restore their sense of significance by shifting their focus and acting extremely within another life domain (e.g., career).

Grounded in social-motivational psychology, Significance Quest Theory (SQT; Kruglanski et al., 2022) posits that all humans have a need for social significance—the feeling that they are important, that they are worthy of respect, and that they matter to others in their social network. The need for significance must be met according to the reality shared with others (Higgins, 2019), meaning that people can gain significance by serving culturally shared values and norms that are valued by their reference group (Webber and Kruglanski, 2016; Kruglanski et al., 2022).

According to the SQT, the magnitude of one’s quest for significance differs both between individuals and within individuals. That is, the need for significance can represent a chronic personality feature, with some having a stronger need for significance than others, but can also be situationally activated. The quest for significance can be activated by experiencing *significance loss* feelings or by perceiving opportunities for *significance gain* (Kruglanski et al., 2014). For example, experiences of humiliation (Brown and Dutton, 1995; Otten and Jonas, 2014) can damage individuals’ sense of mattering (Elliott et al., 2004) and ego-security (Downey et al., 1994; Feldman and Downey, 1994). Also, intense emotions originating from social exclusion (Baumeister et al., 1993), or failure (Brown and Dutton, 1995) can induce significance loss feelings, thereby activating one’s quest for significance. When the quest for significance is activated, for instance through a loss in their sense of significance, they strive to restore their sense of dignity and mattering.

One of the key assumptions of the SQT entails the *substitutability* of means through which one’s sense of significance can be attained. That is, the theory predicts that when people’s sense of significance is threatened with respect to some context, they could be motivated to restore their sense of mattering through substitute actions implemented within *alternative contexts*. For example, if a person is left by their romantic partner, surely a loss of significance, they will be motivated to restore their sense of significance through actions that match cultural cherished values, for instance, through professional success, thereby focusing their energy on their career.

The possibility that individuals can resolve their loss of significance through actions that are substitutable for each other has been anticipated by a large amount of evidence in social motivational psychology (e.g., Wicklund and Gollwitzer, 1981; Steele, 1988; Solomon et al., 1991; Sherman and Cohen, 2006). For instance, research grounded in symbolic self-completion theory (Wicklund and Gollwitzer, 1981) showed that those who feel incomplete regarding a self-defining goal restore their completeness through symbols (i.e., replacement elements) related to that goal. As another striking example, terror management research demonstrated that when people are reminded of morality, they restore their sense of safety by defending their cultural worldviews (for a review, see Greenberg et al., 2014).

As described above people must adhere to *socially cherished values* to restore their sense of significance. One such cherished value through which one can restore their significance is love (Contu et al., 2023). There are at least three reasons why love can confer social significance. First, being loved by a romantic partner make one feel worthy of affection, thereby directly enhancing one’s sense of significance. The human quest for love and attention, in fact, begins in infancy (Bowlby, 1979). Second, providing love to an amorous partner is likely to result in their reciprocation of love and appreciation, which, in turn, furnishes a sense of significance. Lastly, having a romantic relationship responds to culturally shared standards (Baumeister and Leary, 1995; Roberts and Robins, 2000), especially when one’s romantic partner is respected (i.e., validated) by one’s social network. Thus, having an amorous relationship, particularly one with a socially significant other, bestows a sense of significance.

Love does not stand alone in this respect, however. One’s professional life can also be a significance-affording domain. Beyond bestowing a material livelihood, one’s career can inform one’s identity, create relationships, and convey a sense of purpose (Andreassen, 2013). One’s career can similarly enhance one’s significance by granting one a sense of agency and independence as well as a sense that one has made a positive contribution to society. Indeed, many cultures measure life success through professional achievements (Parker and Chusmir, 1992).

Once an individual finds a life domain which they believe will provide them with social significance, they will tend to act extremely within that context when their need for significance becomes dominant. Following this logic, significance quest theory (Kruglanski et al., 2022) has been widely used to explain extreme behaviors and attitudes (Kruglanski et al., 2019). Accordingly, scholars have repeatedly found that when a person is driven by the need to renew their sense of significance (e.g., they have been humiliated), they are disposed to enact extreme behaviors (e.g., self-sacrifice) in pursuit of culturally-valued causes to gain respect from their network (Routledge and Arndt, 2008; Kruglanski et al., 2009; Olivola and Shafir, 2013; Dugas et al., 2016; Kruglanski et al., 2019). All the above-mentioned research, however, linked the personal quest for significance with extremism specifically in the forms of political activism or terrorism (Kruglanski et al., 2013). Expanding this knowledge, Resta et al. (2022) found that ambitious people, who have a strong need for significance, were prone to extremism via obsessive (but not harmonious) passion (Vallerand et al., 2003). Further, Contu et al. (2023) reported that people experiencing general feelings of significance loss were disposed to act extremely within their romantic relationships via obsessive (but not harmonious) romantic passion (Ratelle et al., 2013).

### The present research

In summary, individuals focus their search for significance within socially valued contexts (e.g., romantic relationships, work, in-group’s ideologies). Along this line, psychological states similar to that of loss of significance, and *general* significance loss feelings have been linked to extreme behaviors (e.g., self-sacrifice) in support of a cherished cause (Kruglanski et al., 2009; Dugas et al., 2016). Moreover, *general* feelings of significance loss have been already related to obsessive relational intrusion and readiness to self-sacrifice (i.e., extremism) in romantic relationships (Contu et al., 2023). However, to our knowledge, With the exception of important research regarding political violence (e.g., Kruglanski et al., 2020), the possibility that the motivational imbalance consequent to a situation of significance loss can *shift* from its originating life domain to another life domain in which extremism is enacted is yet unstudied.

Aiming to address this gap in knowledge, we tested whether experiencing a loss of significance originating in the romantic domain can bring people to act extremely in the professional domain (Study 1). We also tested the opposite path, whether experiencing a lack of significance originating in the professional domain, can lead the individual to become obsessive and intrusive (Cupach and Spitzberg, 1998) toward their romantic partner (Study 2). Moreover, in both studies, we controlled for participants’ gender, age, sexual orientation, and relationship duration.

## Study 1

The findings from prior research described above make clear that *general* feelings of significance loss can bring people to act extremely within cherished and valuable life domains (Dugas et al., 2016; Kruglanski et al., 2019; Contu et al., 2023). However, it remains to be determined if experiencing significance loss related to a specific and important life domain (e.g., romantic life) can result in engaging in extreme behavior within an alternative but nevertheless important life domain (e.g., the professional life). Thus, in Study 1, we tested the hypothesis that significance loss feelings related to one’s romantic life can bring people to act extremely at work. Importantly, we also tested if our hypothesis remained consistent after controlling for variables that could have played an important role in this process (i.e., age, gender, relationship duration, and sexual orientation).

### Method

#### Participants, design, and procedure

We made an *a-priori* estimation of the necessary sample size to test our linear multiple regression models through the software G*Power 3.0 (Faul et al., 2007). Assuming small to medium effect sizes (*f^2^* = 0.10), the power set at 0.80, and five predictors, G*Power indicated a minimum sample size of at least 134 participants.

To test our hypothesis, we enrolled 137 Italian adults (18.2% males; *M*_age_ = 34.71, SD_age_ = 7.42) in a correlational study. Given the nature of our hypotheses, we included only participants who had an ongoing romantic relationship (4.4.% homosexual) and were employed. The average duration of participants’ relationships was 110.62 months with an SD = 84.71. Regarding the nature of participants’ romantic relationships, 3.6% of participants were dating, 54.0% were in a stable relationship, 2.9% were engaged, and 39.4% were married. Participants were contacted on social media (e.g., Telegram, Facebook) through an online procedure provided by Google Moduli. After giving their informed consent, each participant filled out an online questionnaire aimed at assessing basic demographic information (i.e., age, gender, relationship duration, and sexual orientation), and the measures of interest as listed below.

#### Measures

##### “Romantic” loss of significance

Feelings of significance loss originating in the context of participants’ romantic relationships were assessed with a five-item measure adapted from Contu et al. (2023). Participants responded on a seven-point Likert scale ranging from 1 (do not agree at all) to 7 (very strongly agree). Examples of those items are: “My relationship makes me feel humiliated” and “My relationship makes me feel disrespected.” Reliability was good (α = 0.89).

##### Extremism at work

Proneness to act extremely at work was assessed through an Italian version of the Extremism Scale adapted to the professional domain. Participants were asked to indicate the extent to which their life was dominated by having professional success (12 items, e.g., “I usually invest all of my time and energy to have professional success”). Items were rated on a 7-point Likert scale ranging from 1 (Definitely disagree) to 7 (Definitely agree). Reliability was excellent (α = 0.95).

#### Data analysis

To test our hypothesis, we tested a multiple linear regression model in which significance loss feelings originating in the romantic context, participants’ gender, age, relationship duration, and sexual orientation were the independent variables, and work-related extremism was the examined outcome. Analyses were conducted using SPSS Statistic version 27.0 and bias-corrected confidence intervals were obtained with 5,000 bootstrap samples (descriptive statistics are displayed in Table 1).

**Table 1:** Study 1.

	1	2	3	4	5	6	*M* (SD)
1. LOSS_c	(0.89)	0.162	0.187*	0.121	0.162	0.13	1.18 (1.12)
2. AGE	–	–	−0.060	0.735***	−0.039	0.161	34.71 (7.42)
3. EXTR_w	–	–	(0.95)	−0.125	−0.163	0.029	2.44 (1.31)
4. DUR	–	–	–	–	0.138	0.211*	110.62 (84.71)
5. GENDER	–	–	–	–	–	−0.009	–
6. OR	–	–	–	–	–	–	–

*N* = 137. Bivariate correlations and descriptive statistics.

*p < 0.05. **p < 0.01. ***p < 0.001. LOSS_c = Relationship-related significance loss, AGE = Participants’ age, OR = Sexual orientation (coded as 1 = homosexual; 2 = heterosexual), EXTR_w = Extremism at work, DUR = Relationship duration, GENDER coded as 1 = males; 2 = females. In bracket (Cronbach’s Alpha).

### Results

#### Multiple regression analysis

Results of the multiple linear regression model revealed that feelings of significance loss originating in participants’ romantic relationships positively and uniquely predicted participants’ tendency to act extremely at work [*b* = 0.24, SE = 0.01, *t* = 2.73, *p* = 0.007 (95%CI = 0.07, 0.55)]. Participants’ tendency to act extremely at work was not predicted by any of the other variables we considered (age, *p* = 0.83; gender, *p* = 0.06; relationship duration, *p* = 0.38; participants’ sexual orientation, *p* = 0.47). The entire model was significant [*F*(5, 130) = 2.63, *p* = 0.027, *R*^2^ = 0.06].

### Discussion

In Study 1, we investigated the relationship between romance-related significance loss feelings and extremism at work. Particularly, we aimed to explore whether individuals who are feeling insignificant because of their romantic relationship are more disposed to extremism at work while controlling for variables that could have played an important role in this process (i.e., age, gender, relationship duration, and participants’ sexual orientation). Findings corroborated our hypothesis. Indeed, participants experiencing loss of significance related to their romantic relationship were more prone to extremism at work. Importantly, among all the variables we included in the model as predictors, results showed that only significance loss within the romantic context was a significant predictor of extremism at work.

These results suggest that when people experience feelings of loss of self-worth, importance, dignity, and respect related to the specific context of their romantic relationships, they can restore their sense of significance by engaging in extreme behavior in another important life domain (i.e., professional life). That is, these results directly support the SQT’s key assumption that individuals can resolve their lack of significance through actions that are substitutable for each other (Kruglanski et al., 2022).

## Study 2

The results of Study 1 were consistent with our hypothesis. We thus conducted a second study to bolster our hypothesis and to extend our findings by testing the path opposite to that analyzed in the first study. Indeed, if people experiencing significance loss feelings related to the romantic context are more prone to extremism at work, then the reverse should also be true. Thus, Study 2 tested the hypothesis that people experiencing work-related significance loss feelings should be more disposed to act extremely toward their romantic partner. Given their importance within societies and cultures, romantic relationships, like professional success, should be a fertile ground where people can seek social significance. As in Study 1, we also tested if our hypothesis remained consistent after controlling for variables that could have played an important role in this process (i.e., age, gender, relationship duration, and participants’ sexual orientation).

Notably, in this second study, our examined outcome (i.e., extreme behavior) was obsessive relational intrusion (ORI; Cupach and Spitzberg, 1998). Explicitly, ORI has been defined as the repeated and undesired pursuit and invasion of one’s partner’s privacy. For example, ORI could include insisting that one’s partner not spend time with other people or demanding that one’s partner share their passwords for their personal devices. This specific type of extreme behavior is of particular interest because of its serious consequences for its victims. For example, being a victim of ORI is associated with poor health and high levels of depressive symptoms (Davis et al., 2002), as well as physiological, mental, and social disorders (Nguyen et al., 2012). Further, high levels of post-traumatic stress disorder have been found among victims of ORI (Basile et al., 2004), which showed also strong correlations with psychological maltreatment (Davis et al., 2000).

### Method

#### Participants, design, and procedure

We used the same sample size determination strategy employed in Study 1. To test our hypotheses, we thus enrolled 148 Italian adults in a correlational study (35.8% males, *M*_age_ = 35.18, SD_age_ = 11.18). As in Study 1, we included only participants who had an ongoing romantic relationship (4.1.% homosexual) and were employed. The average duration of participants’ relationships was 120.55 months (SD = 119.07). Moreover, 5.4% of participants were dating, 4.1% were in a stable relationship, 37.8% were engaged, and 52.7% were married. The procedure was the same as that used in Study 1.

#### Measures

##### Work-related significance loss

We measured work-related significance loss by adapting items from Study 1. Examples of those items are: “My work makes me feel humiliated” and “My work makes me feel disrespected.” Reliability was good (α = 0.80).

##### Obsessive relational intrusion

Participants completed an abridged version of the ORI scale composed of 20 items (Cupach and Spitzberg, 2004), measuring participants’ tendency to engage in intrusive behaviors in order to maintain their relationship. Participants responded on a seven-point Likert scale ranging from 1 (absolutely not) to 7 (absolutely yes). Example items include: “To maintain my relationship I would be willing to spy on my partner’s cell phone” and “To maintain my relationship I would be willing to threaten to harm myself.” Reliability was good (α = 0.89).

#### Data analysis

To test our hypothesis, we tested a multiple linear regression model in which work-related significance loss feelings, participants’ gender, age, relationship duration, and sexual orientation were the independent variables, and obsessive relational intrusion within couples was the examined outcome. Analyses were conducted as in Study 1 (descriptive statistics are displayed in Table 2).

**Table 2:** Study 2.

	1	2	3	4	5	6	*M* (SD)
1. LOSS_w	(0.80)	−0.151	0.218**	−0.120	0.051	−0.056	1.91 (1.05)
2. AGE	–	–	−0.136	0.825***	−0.027	0.000	35.18 (11.18)
3. ORI_c	–	–	(0.89)	−0.080	−0.083	−0.043	1.81 (0.72)
4. DUR	–	–	–	–	0.034	0.075	120.55 (119.71)
5. GENDER	–	–	–	–	–	0.060	–
6. OR	–	–	–	–	–	–	–

*N* = 148. Bivariate correlations and descriptive statistics.

*p < 0.05. **p < 0.01. ***p < 0.001. LOSS_w = Work-related significance loss, AGE = Participants’ age, OR = Sexual orientation (coded as 1 = homosexual; 2 = heterosexual), ORI_c = Obsessive relational intrusion, DUR = Relationship duration, GENDER coded as 1 = males; 2 = females. In bracket (Cronbach’s Alpha).

### Results

#### Multiple regression analysis

Results of the multiple linear regression model revealed that work-related significance loss feelings positively predicted participants’ tendency to engage in obsessive relational intrusion toward their romantic partner [*b* = 0.21, SE = 0.06, *t* = 2.54, *p* = 0.01 (95%CI = 0.03, 0.28)]. Participants’ tendency to engage in obsessive relational intrusion toward their partner was not predicted by any of the other variables we considered (age, *p* = 0.18; gender, *p* = 0.22; relationship duration, *p* = 0.40; participants’ sexual orientation, *p* = 0.80). The entire model was significant [*F*(5, 141) = 2.33, *p* = 0.045, *R*^2^ = 0.08].

### Discussion

Results from Study 2 supported and expanded upon those of the Study 1. Results revealed that the opposite path used in Study 1 was also significant; significance loss related to the professional context was positively associated with the tendency to engage in extreme behaviors in the romantic context, this time operationalized as obsessive relational intrusion. The findings thus reinforce the substitutability of means through which one can restore their significance in instances of significance loss. Significance lost in the romantic domain can be restored via extremism in the professional domain and significance lost in the professional domain can be restored via extremism in the romantic domain.

## General discussion

Drawing on Significance Quest Theory (Kruglanski et al., 2022), we hypothesized that people experiencing significance loss feelings related to a specific life domain can try to restore their sense of significance by acting extremely within another similarly important life domain. We based our hypothesis on the SQT’s key assumption entailing the *substitutability* of means through which one’s sense of significance can be attained. That is, the theory posits that when people’s sense of significance is threatened with respect a specific context (e.g., romantic relationships), they could be motivated to restore their sense of mattering through substitute actions implemented within *alternative contexts* (e.g., the workplace).

We tested these hypotheses across two studies, both employing samples of people who were both employed and engaged in a romantic relationship. Study 1 tested if people experiencing significance loss feelings originating in the romantic relationship domain were more prone to extremism at work. Following this logic, Study 2 tested the opposite path. Specifically, Study 2 tested whether people experiencing work-related significance loss were more prone to engage in obsessive relational intrusion (ORI) toward their romantic partner. Results from the two studies corroborated our hypothesis. Indeed, in both studies, significance loss feelings were significantly and positively associated with extremism at work (Study 1) and obsessive relational intrusion (Study 2), while controlling for participants’ age, gender, sexual orientation, and relationship duration.

Interestingly, these results illuminate a process in which significance loss feelings originating in life domains that are expected to be important sources of significance (e.g., career, romantic relationships) shift from one life domain to another, thereby pushing individuals to act extremely in life domains which are not directly connected with the origin of the experience of significance loss. Thus, these results suggest that, since having a stable romantic relationship and professional success are well-established sources of social significance (Bowlby, 1979; Parker and Chusmir, 1992; Baumeister and Leary, 1995; Roberts and Robins, 2000), maintaining one’s relationship or job should be considered fruitful ways to renew one’s sense of significance, even if the loss of significance originated elsewhere.

### Theoretical implications

This research represents one of the first empirical tests of the SQT assumption of *substitutability* of means through which one’s sense of significance can be attained. Also, these results provide further evidence for the theory of motivational imbalance (Kruglanski et al., 2021) by demonstrating that a motivational imbalance (i.e., dominant significance loss feelings) related to a specific life domain can motivate people to act extremely in order to meet their dominant need and restore balance, even within a life domain unrelated to the original source of the imbalance. Moreover, results from both studies furnish additional evidence in support of the notion that love, amorous relationships, and romantic partners are perceived as fruitful in maintaining or restoring ones’ general sense of personal significance, similarly to one’s professional success. These findings are in accordance with a large amount of evidence and other theories beyond SQT and that of motivational imbalance. As mentioned in the introduction, the possibility that individuals can resolve their lack of significance through actions that are substitutable for each other has been widely anticipated (e.g., Wicklund and Gollwitzer, 1981; Steele, 1988; Solomon et al., 1991; Sherman and Cohen, 2006). However, our results seem to be the first direct test of such a possibility within the theoretical frame of the SQT (Kruglanski et al., 2022).

### Limits and future directions

Given the correlational nature of our studies, we were not able to provide evidence about the casual relationship between the constructs we studied in this research. Also, given the cross-sectional approach implemented in both Studies 1 and 2 we were not able to rule out some important factors as possible alternative explanations for the observed effects such as emotional intelligence (Goleman, 1995) or social abilities (Beauchamp and Anderson, 2010), among other potential factors. Further, another limitation of our correlative studies was that we evaluated only self-reported levels of significance loss feelings; thus, we could not investigate the effect of situationally induced feelings of low significance. Hence, future research should address these limitations by implementing true experiments, in which extreme behaviors in the professional and romantic contexts are measured following manipulation of significance loss in the romantic and professional contexts, respectively. In this respect, a recent study carried out by Contu et al. (2023) provided evidence in support of the consequentiality among general (i.e., not related to a specific life domain) significance loss and obsessive relational intrusion toward one’s romantic partner, suggesting that the results of the experiments suggested above would likely support the causal hypothesis. As another limitation, our research considered only two significance-granting life domains, both of which are valued by most Western cultures (e.g., Parker and Chusmir, 1992; Baumeister and Leary, 1995) but are not necessarily prioritized in cultures where, for instance, arranged marriages are common or individual professional success is subservient to collective family honor. Future research should expand upon this knowledge by testing whether significance loss feelings related to other life domains, particularly those valued in non-Western cultures, can provoke extremism within other substitute life domains. Future research should also consider not only the effect that significance loss feelings could have on the tendency to act extremely, but whether significance loss feelings can have different effects depending on their duration. Indeed, although our research confirmed that significance loss feelings are related to the tendency to act extremely within a different life domain, it is possible that the longer one experiences significance loss in a specific life domain, the more likely they will be to engage in extreme behaviors in an alternative life scope. In other words, increased duration of significance loss feelings could serve as a catalyst in the process that brings people from experiences of significance loss feelings to extreme behaviors.

## Data availability statement

The raw data supporting the conclusions of this article will be made available by the authors, without undue reservation.

## Ethics statement

The studies involving human participants were reviewed and approved by the Ethics Committee of Department of Social and Developmental Psychology at “La Sapienza” University of Rome (protocol N. 572). The patients/participants provided their written informed consent to participate in this study.

## Author contributions

FC, ME, AK, and AP contributed to the study conception and design. Material preparation, data collection and analysis were performed by FC, AP, and AK. The first draft of the manuscript was written by FC. Reviews and editing were provided by ME, and all authors commented on previous versions of the manuscript. All authors contributed to the article and approved the submitted version.

## Conflict of interest

The authors declare that the research was conducted in the absence of any commercial or financial relationships that could be construed as a potential conflict of interest.

## Publisher’s note

All claims expressed in this article are solely those of the authors and do not necessarily represent those of their affiliated organizations, or those of the publisher, the editors and the reviewers. Any product that may be evaluated in this article, or claim that may be made by its manufacturer, is not guaranteed or endorsed by the publisher.
